# Neurovascular development and links to disease

**DOI:** 10.1007/s00018-013-1277-5

**Published:** 2013-03-12

**Authors:** Christiana Ruhrberg, Victoria L. Bautch

**Affiliations:** 1 UCL Institute of Ophthalmology, University College London, 11-43 Bath Street, London, EC1V 9EL UK; 2 Department of Biology, The University of North Carolina at Chapel Hill, Chapel Hill, NC 27599 USA; 3 McAllister Heart Institute, The University of North Carolina at Chapel Hill, Chapel Hill, NC 27599 USA; 4 Lineberger Comprehensive Cancer Center, The University of North Carolina at Chapel Hill, Chapel Hill, NC 27599 USA

**Keywords:** Neurovascular, Neural tube, Retina, Hindbrain, VEGF, Semaphorins, Neuropilin, GPCR124, TGFβ

## Abstract

The developing central nervous system (CNS) is vascularized via ingression of blood vessels from the outside as the neural tissue expands. This angiogenic process occurs without perturbing CNS architecture due to exquisite cross-talk between the neural compartment and invading blood vessels. Subsequently, this intimate relationship also promotes the formation of the neurovascular unit that underlies the blood–brain barrier and regulates blood flow to match brain activity. This review provides a historical perspective on research into CNS blood vessel growth and patterning, discusses current models used to study CNS angiogenesis, and provides an overview of the cellular and molecular mechanisms that promote blood vessel growth and maturation. Finally, we highlight the significance of these mechanisms for two different types of neurovascular CNS disease.

## Introduction

The vertebrate central nervous system (CNS), comprised of brain, spinal cord, and retina, is vascularized during its development to provide oxygen and nutrients to newly born neurons, long before they extend axons and dendrites. The neural tube acquires its own vascular network prior to birth via angiogenic sprouting from vessel networks that form immediately outside the CNS; the intraneural blood vessel network then expands as the neural tissue grows. In contrast, the multilayered retina is initially supplied by a combination of two extra-retinal vascular systems, the choroidal vasculature that supplies the outer retina, and the hyaloid arteries that supply the inner retina and lens; the choroidal vasculature persists, but late in mammalian development, the hyaloid arteries are replaced with a dedicated intraretinal vascular system. In this review, we will provide a historical perspective on research into neural tube and retinal angiogenesis, discuss current models available to study CNS angiogenesis, and summarize recent progress in uncovering the cellular and molecular mechanisms of blood vessel growth and maturation in the CNS.

## Description of neurovascular development

### Historic perspective

The process of CNS vascularization was first studied in the fetal chick brain and both rat and rabbit cerebral cortex with a combination of India ink perfusion and electron microscopy to reveal the structure of patent blood vessels [1–3]. In combination with conventional histological techniques, these studies revealed that blood vessels first form a perineural vascular plexus (PNVP), and then invade and branch within the neural tube in stereotypical patterns. Subsequent electron microscopy studies of spinal cord vascularization in the developing mouse embryo demonstrated interactions between endothelial cells and neural cells, and suggested that both cell types contribute to the blood–brain barrier (BBB) [4].

Following on from the pioneering studies, subsequent research into the mechanisms of CNS vascularization used antibody-based and embryological techniques to trace the behavior of developing blood vessels in the neural tube. Initially, the QH1 antibody, which recognizes quail angioblasts and endothelial cells [5], was used to analyze avian vascularization [6]. QH1 staining allowed researchers to follow the fate of quail endothelial cells after transplantation into chick hosts and provided strong evidence that the CNS was vascularized by angiogenic sprouting [7–9]. More recently, staining for QH1 and in situ hybridization have been combined with neural tube electroporation to selectively manipulate one side of the avian neural tube, with the contra-lateral side serving as an internal control [10, 11].

Since the discovery of QH1, additional vascular markers have become available that facilitate the study of brain and retinal angiogenesis in other vertebrates, including mouse. Accordingly, immunohistochemical, immunofluorescent, and immunoblotting techniques are now commonly combined with traditional methods to study the molecular and cellular mechanisms of vessel growth in the CNS. In particular, immunological techniques have been used to compare CNS angiogenesis in normal development and after genetic modification of candidate vascular growth and patterning factors.

**Fig. 1:**
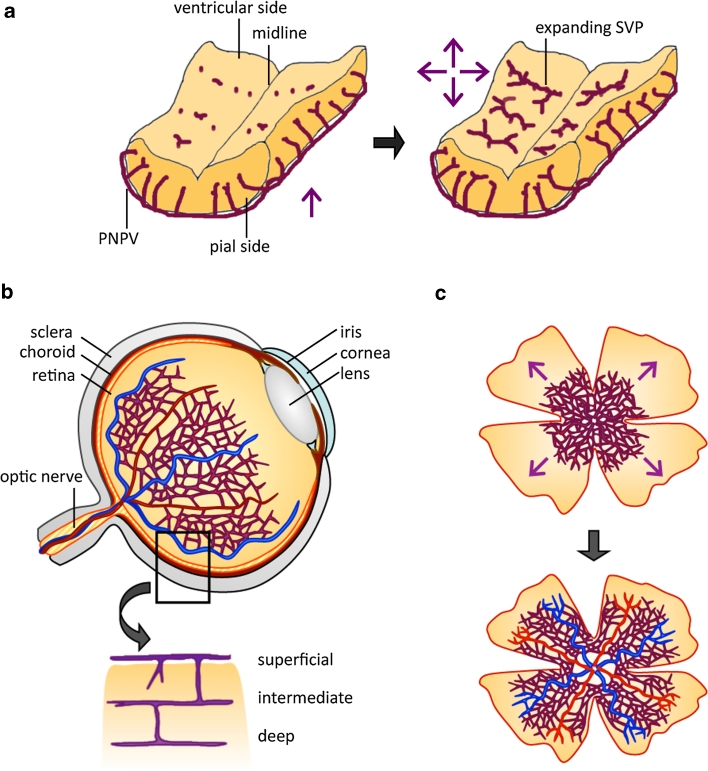
Time course of blood vessel ingression into the mouse embryo hindbrain and the perinatal mouse retina. **a** Vessels sprout from the PNVP into the hindbrain at around embryonic day 9.75 in the mouse and then grow radially towards the ventricular zone. Radial vessels do not invade the subventricular zone, but sprout laterally and then anastomose to form a subventricular vascular plexus by E12.5. **b** Cross section of an adult eye shows the relationship of retinal vessels to other ocular structures (*top half*) and the subdivision of the retinal vasculature into three plexi, termed superficial (or primary plexus), intermediate plexus and deep plexus. **c** Retinal vascularization proceeds from center to periphery in a radial fashion during the first week of life (*upper half*) and leads to an extensively remodeled superficial plexus (*lower half*)

Not long after the discovery of brain vascularization through angiogenic vessel ingression, it was shown that a comparable mechanism also operates in the mammalian retina, with angiogenic vessel ingression from the optic nerve vasculature [12, 13]. Currently, the perinatal mouse retina is the most widely studied model system for studying CNS vascularization (reviewed in [14]), followed by the mouse embryo hindbrain [15] (Fig. 1). The popularity of these models results from the genetic tractability of the mouse embryo, the ever-increasing availability of antibodies for relevant mouse proteins, and the ability to perform quantitative studies on flat-mounted tissues. A model system that complements and diversifies the use of immunolabeled mouse tissues is the zebrafish embryo, because several fluorescent transgenic reporters for endothelial cells have been developed for live imaging of vascular development [16, 17]. For example, transgenic fish that link GFP or mCherry to vascular promoters such as *kdrl* (*flk1*) or *fli1* are used extensively, and can be used to study CNS vascularization in normal and genetically altered fish embryos.

### Description of CNS vascularization: hindbrain and retina models

The vascularization of the mouse embryo hindbrain is initiated around embryonic day (E) 9.5, when a few vascular sprouts emerge from the PNVP and invade the hindbrain parenchyma (Fig. 1a, left-hand side; e.g., [18]). At E10.25, these radially growing vessels begin to sprout at near right angles and extend parallel to the hindbrain surface that faces the fourth ventricle (Fig. 1a, right-hand side). As sprouts from neighboring radial vessels meet and anastomose, the subventricular vascular plexus (SVP) is formed (Fig. 1a, right-hand side) [15, 18]. This vascular fusion process is promoted by yolk sac-derived tissue macrophages, which interact with endothelial tip cells and thereby act as bridge cells between neighboring vessel sprouts [18]. By E12.5, an extensive vascular network has been established in the hindbrain, consisting of radial vessels originating from the perineural plexus and the SVP that is placed orthogonally to the radial vessels [15]. Following on from these early stages, sprouting and fusion moves to deeper brain layers, but the precise events that drive this process are not yet understood.

Vascularization of the avian neural tube occurs in a similar manner [1, 9, 10]. At the limb level, the PNVP forms between Hamburger Hamilton (HH) stages 16 and 24 (E2.5–4.5) in the quail, with ingression beginning at HH stages 22–24 (E4–4.5). The ingression points are not exact, but cluster around a ventral and more lateral point on each side of the midline [10]. The ingressing vessels migrate dorsally or medially until they reach the subventricular zone, where they branch at right angles, extend, and anastomose to enable circulation [1, 4]. Early work suggested that brain vascularization in the chick does not involve angioblastic single cell precursors [19]. However, QH1 labels single, ramified cells in the quail dorsal neural tube that interact with nascent vessels [9]. These cells were originally proposed to be angioblasts, but were subsequently shown to be macrophages that interact with vascular endothelium [20] and likely correspond to the tissue macrophages that promote the fusion of neighboring vessel sprouts in the mouse embryo hindbrain [18].

In contrast to the hindbrain and neural tube, the retina is vascularized only after birth in rodents to give rise to a system of three vascular plexi that are interconnected (Fig. 1b; reviewed in [14]). Vascularization begins on the day of birth, when vessel sprouts emerge from the optic nerve head and spread radially over the retina, guided by a template of astrocytes (Fig. 1c), with blood vessels and astrocytes forming co-patterned networks [21–23]. During the process of radial expansion, the primary plexus undergoes arteriovenous differentiation (Fig. 1c; reviewed by [14]). After the first week of life, vessel sprouts emerge from this primary retinal vessel plexus to dive into the inner retinal layers at near right angles and form the deep plexus in week two and then the intermediate plexus in week three after birth (Fig. 1b, lower half; reviewed by [14]). While the contribution of astrocytes to primary plexus formation has been extensively studied, the cellular scaffolds that guide vessel sprouting into the deeper retinal layers are still poorly defined. As observed in the hindbrain, vascular anastomosis of retinal blood vessels is promoted by macrophages [18, 24–26]. Retinal macrophages also contribute to subsequent vascular remodeling in the retina. Thus, the decrease in initial vascular network complexity is compensated for by pruning of fewer vessel segments at later developmental stages in *Csf1* ^*op/op*^ mutants with defective macrophage recruitment, or *Pu1* ^*−/−*^ mutants lacking macrophages [18]. Consequently, the adult retinal vasculature reaches normal complexity in *Csf1* ^*op/op*^ mutants that survive to adulthood [24].

Due to the increasing availability of useful markers, precise genetic mutations in proteins regulating blood vessel growth, the planar orientation of sprouting blood vessels and the proximity of the emerging vessel plexus to the tissue surface, both the mouse hindbrain and retina models allow excellent visualization of vessel growth. It is therefore not surprising that these CNS regions have replaced the rat and rabbit cortex as preferred models to study CNS vascularization. However, not all vertebrates have a retinal vasculature [13, 27], and certain aspects of vessel patterning may be unique to the cortex. Accordingly, one study provided evidence that cell autonomous programs regulated by *Hox* genes lead ventral sprouts to colonize dorsal areas of the telencephalon, rather than sprouting from the dorsal PNVP [28].

An emerging model of neurovascular development is the zebrafish, which is particularly amenable to rapid genetic manipulation and longitudinal live imaging [17]. Two recent studies have described the process of hindbrain vascularization in the zebrafish embryo [29, 30]. The spatial relationship of vessel ingression sites and rhombomere boundaries in the zebrafish hindbrain suggests neurovascular cross-talk [30] that appears to be conserved in other vertebrates, although this is less well studied. In this context, it is interesting that rhombomere boundaries in the chick are extracellular spaces rich in growth factor-binding proteoglycans [31, 32].

### Cellular behaviors and interactions in neurovascular development

Like elsewhere in the body, blood vessels in the CNS are comprised of endothelial cells that are invested with mural cells. Although common to other vascular beds, some of the underlying principles that govern cellular interactions of endothelial cells amongst each other and with mural cells were first elucidated using the retina and hindbrain models, such as the tip cell-stalk cell paradigm (reviewed in [33]).

Endothelial tip cells respond to signals by initiating migration, while endothelial stalk cells follow behind the tip cell and respond to signals with proliferation and lumen formation to form the main body of new vascular sprouts. Initial experiments linked tip cell and stalk cell behaviors to signals provided by the vascular endothelial growth factor VEGF-A (referred to as VEGF in the remainder of this review) [15, 34]. Subsequent studies showed that VEGF interacts with the delta like 4 (DLL4)/notch pathway to regulate tip cell vs. stalk cell number [35–37]. Studies of chimeric embryoid bodies and developing retinal vessels suggested that tip cell and stalk cells do not remain fixed, but switch phenotypes over time [38]. Accordingly, the tip and stalk cell phenotypes are plastic states of functional specialization.

Consistent with a key role for VEGF in tip cell induction in the retina and hindbrain in vivo, a high level of VEGFR2 and low level of VEGFR1 relative to neighboring endothelial cells promotes tip cell behavior in chimeric embryoid bodies [38]. Recent work identified additional regulators of vessel sprouting and tip cell behavior, for example BMP signaling [39–41] and SEMA3E signaling through PLXND1 (discussed in more detail below) [42]. Several tip cell markers have also been identified via expression analysis, and their function in CNS angiogenesis is presently being characterized [43, 44].

In addition to the general principles of angiogenesis described above, specialized cellular interactions between endothelial and non-endothelial CNS cells create a unique structure called the neurovascular unit. In this structure, endothelial cells form firm junctions with each other and interact with other cell types to create the BBB; this barrier maintains CNS homeostasis and is also thought to regulate CNS blood flow and synaptic activity [45, 46]. A hallmark of CNS vessels is the expression of the glucose transporter GLUT1. Mutations in the *GLUT1* gene that lead to GLUT1 deficiency cause a rare autosomal dominant disorder characterized by a low cerebrospinal fluid glucose concentration due to reduced transport across the BBB [47].

In addition to endothelial cells, the neurovascular unit contains pericytes, astrocytes, oligodendrocytes, and microglia. Two recent studies showed that loss of pericytes in the CNS elevates endothelial transcytosis [48, 49]. Accordingly, pericyte-endothelial interactions are necessary to maintain the BBB by preventing exchange across the endothelium, complementing the role of tight intra-endothelial cell junctions in preventing paracellular exchange. The molecular cross-talk among the cell types of the neurovascular unit is only partially characterized, but is regulated by TGFβ, PDGF, BMP, and integrins; accordingly, disruption of these signaling axes perturbs the BBB [48–53].

## Key signals regulating CNS angiogenesis

Although many signaling pathways contribute to vascular development in general, we focus here on key pathways that are critical for the cross-talk between the nervous and vascular systems to regulate blood vessel patterning in the CNS.

### VEGF

Several neural cell types produce VEGF, and neuroglial VEGF is required for the ingression of blood vessels into the developing neural tube and retinal vascularization across different vertebrate species (e.g., [10, 29, 54–57]). VEGF is differentially spliced to produce isoforms with a differential affinity for the surrounding extracellular matrix [58], and their bioavailability is further regulated by proteolytic mechanisms [59, 60]. Amongst these isoforms, VEGF121 is the most diffusible, VEGF189 binds the matrix most avidly, and VEGF165 has intermediate properties. Cleavage of the VEGF189 isoforms by matrix metalloproteases leads to the generation of the VEGF113, which is released from the matrix. Whereas the human isoforms are termed VEGF121, VEGF165, and VEGF189, reflecting the number of amino acid residues in the mature protein, the corresponding mouse isoforms are one amino acid residue shorter and therefore termed VEGF120, VEGF164, and VEGF188, respectively.

In the CNS, genetic manipulations that lead to expression of only a single VEGF isoform do not prevent ingression, but affect vessel patterning and morphogenesis. Accordingly, hindbrain vessels in *Vegfa* ^*120/120*^ mice expressing only the VEGF120 isoform have a larger caliber and branch infrequently, while vessels in *Vegfa* ^*188/188*^ mice expressing only VEGF188 are thin and over-branched [15]. In the quail in the neural tube, the localized over-expression of matrix binding VEGF165 or VEGF189, but not VEGF121, also leads to ectopic vessel ingression at the site of over-expression, whilst local VEGF blockade prevents ingression [10].

In the neonatal mouse retina, a collection of the three VEGF isoforms is produced and displayed by an astrocytic network that is located beneath the expanding vascular plexus (e.g. [23, 61]). However, astrocyte-derived VEGF is not essential for the initial vascular growth of retinal vessels, but instead for the survival of nascent retinal vessels [62] and hypoxia-induced neovascularization in a mouse model of oxygen induced retinopathy [63]. While astrocyte-derived VEGF alone is not essential for angiogenic sprouting in the developing retina, the more widespread deletion of VEGF from the neuroretina severely perturbs retinal angiogenesis [55]. It is not yet known if one specific cell type provides an essential VEGF cue, or if multiple cell types provide redundant sources of VEGF.

The tyrosine kinase FLK1 (KDR, VEGFR2) is the main signal transducing VEGF receptor in endothelial cells in vitro and essential for endothelial cell survival and blood vessel formation, with its loss leading to embryonic lethality at E9.5 in the mouse (reviewed in [64]). Due to their early embryonic lethality, *Flk1* knockout mice are not suitable to study the specific roles of FLK1 signaling in CNS vascular development. However, use of a function-blocking antibody revealed that FLK1 is essential for tip cell formation and vascular outgrowth in the retina [34]. FLK1 was originally thought to be a critical mediator of VEGF-A-induced DLL4 expression and signaling in sprouting retinal blood vessels; however, a recent study showed that when *Flk1* is deleted in endothelial cells, retinal angiogenesis depends on the alternative VEGF family receptor tyrosine kinase FLT4 (VEGFR3), which is best known for its role as a VEGF-C receptor in lymphangiogenesis [65].

### Neuropilins, neuropilin-binding VEGF-A isoforms, and semaphorins

Neuropilin 1 (NRP1) is a non-catalytic transmembrane protein whose genetic loss, either globally or specifically in endothelial cells, severely inhibits CNS vascularization, as shown for mouse in the spinal cord [66], hindbrain [67], and forebrain [68]. In contrast, the perisomatic regions, located outside the CNS, are vascularized in the absence of NRP1, with only minor morphological defects [15]. It is not yet known why NRP1 is essential for CNS vascularization, but less important for some other vessel beds. Relevant to CNS vascularization, NRP1 serves as a receptor for VEGF165 and a member of the structurally unrelated class 3 semaphorin family termed SEMA3A (reviewed in [69]). Both ligands bind to distinct NRP1 domains and can therefore bind simultaneously, rather than competitively [70]. VEGF121 can also bind NRP1, but with 50-fold lower affinity than VEGF165, due to the absence of an exon 7-encoded domain that enhances binding [71].

Both VEGF165 and SEMA3A have been implicated as modulators of endothelial cell behavior. However, studies of mouse knockouts lacking SEMA3A have shown that this NRP1 ligand is dispensable for normal brain vascularization and blood vessel formation elsewhere in the developing mouse [72, 73]. In agreement, inactivation of semaphorin binding to NRP1 does not affect brain angiogenesis or vascular development in the early mouse embryo. Moreover, co-ablation of the related neuropilin NRP2, which also serves as a VEGF and SEMA receptor, to abrogate all semaphorin signaling through NRP1 and NRP2, does not affect CNS vascularization [72, 74].

The above studies did not identify roles for SEMA3A in developmental angiogenesis, but tumor studies implicated SEMA3A as a modulator of pathological angiogenesis. Thus, SEMA3A reduces the overall vascularity of tumors and “normalizes” tumor vessels, in part by recruiting myeloid cells that stimulate vessel maturation [75]. Moreover, high concentrations of SEMA3A evoke vascular permeability in the skin of adult mice by stimulating signaling through plexin-NRP1 complexes [76], which are better known for their role in neural guidance during development (reviewed in [69]). Whether these findings on the role of SEMA3A in the adult vasculature are also relevant to CNS blood vessels has not yet been examined.

Semaphorin signaling through NRP1 does not impair brain vascularization, but loss of NRP1 from endothelial cells causes vascular brain defects similar to those caused by loss of NRP1 in all cell; accordingly, it was proposed that VEGF-A rather than SEMA3A signaling through NRP1 is essential for vascular development [68]. Yet, direct evidence that NRP1 serves as a VEGF164 receptor in angiogenesis is still lacking. In this context, it is interesting to note that *Vegfa* ^*120/120*^ mice lacking heparin/neuropilin binding VEGF isoforms have milder CNS vascular defects than mice lacking NRP1 [15, 67]. This observation raises the possibility that NRP1 promotes brain angiogenesis through additional, but as yet unidentified, signaling mechanisms. In vitro, NRP1 interacts with a host of additional proteins, including several growth factors and adhesion molecules [77–81]. Which of these interactions are physiologically relevant to CNS vascularization remains to be determined.

SEMA3E is the only class 3 semaphorin that does not bind to a neuropilin receptor, but instead binds directly to the plexin PLXND1 [74]. In the developing retinal vasculature, high VEGF levels emanating from the avascular retinal periphery induce PLXND1 expression in endothelial cells at the vascular front in a VEGFR2-dependent manner [82]. Loss-of-function studies further demonstrated that SEMA3E, derived from the neural layers of the retina, signals through endothelial PLXND1 to upregulate DLL4 at the vascular front, which in turn increases endothelial Notch signaling to a loss of tip cells and tip cell filopodia [82]. Consequently, normal vascular expansion into the retinal periphery is disrupted in mice lacking SEMA3E [82]. Even though SEMA3E does not directly bind to NRP1, in CNS neurons NRP1 can convert SEMA3E/PLXND1-mediated axonal repulsion into attraction [83]. Whether similar mechanisms operate in endothelial cells to modulate SEMA3E signaling is not known. Remarkably, SEMA3E normalizes VEGF-A-induced pathological vessel growth in a mouse model of oxygen-induced retinopathy, in which retinal vessels grow abnormally into the vitreous [84]. Thus, the intravitreal administration of SEMA3E protein prevented this abnormal vessel growth and instead normalized vessel growth within the retina. This observation makes SEMA3E a potential therapeutic tool to fine tune VEGF-A signaling and vascular growth in the ischemic nervous system.

### Other neurovascular signals

Several additional signaling pathways have recently been identified as important for CNS angiogenesis, with roles in blood vessel ingression and/or survival. The genetic loss of both WNT7a and WNT7b reduces neural tube angiogenesis, and utilizes the canonical Wnt/β-catenin pathway, as vascular-specific loss of β-catenin resulted in reduced neural tube vessels, and those that ingressed were dilated and hemorrhagic [85, 86]. Effectors of Wnt signaling in CNS angiogenesis may include the death receptors DR6 and TROY, which are downstream transcriptional targets of Wnt/β-catenin signaling that are also required for proper brain angiogenesis and BBB formation [87].

Several studies revealed a requirement for the orphan G protein coupled adhesion receptor GPCR124 in endothelial cells for proper neural tube vascularization [88–90]. Specifically, loss of GPCR124 in mice delays but does not completely prevent blood vessel ingression. Moreover, abnormal glomeruloid tufts and hemorrhages are associated with defective BBB formation in the absence of GPCR124. Interestingly, GPCR 124 is downstream of TGFβ, whose loss has also been linked to defective CNS angiogenesis and a compromised neurovascular unit [91]. Finally, mouse knockout studies showed that αvβ8 integrin expression by neural, but not endothelial cells is required for normal CNS vascularization and to prevent hemorrhage at later embryonic and postnatal stages [51, 52, 92]. Because integrins have been shown to act upstream of TGFβ signaling, it appears likely that integrin expression in neural cells stimulates TGFβ signaling in endothelial cells, which initiates GPCR124 expression for proper neurovascular development.

## Neurovascular diseases

The many facets of neurovascular disease have recently been reviewed extensively [93]. Here, we discuss the recent progress in identifying the molecular and cellular mechanisms of two types of brain disease, cerebral cavernous malformations (CCMs) and brain cancer.

### CCM

Cavernous hemangiomas in the brain consist of thin-walled, fragile blood vessels with poor blood flow that are known as CCMs. They are caused by mutations in proteins that are important for cerebral vascular integrity (reviewed in [94, 95]). Three distinct heterozygous familial mutations in CCM1 (KRIT1), CCM2 (MGC4607, OSM), or CCM3 (PDCD10) predispose to CCM formation, with a second, somatic hit likely leading to loss of heterozygosity and disease manifestation [96]. Sporadic CCM has also been linked to these three genetic loci. Recent work shows that CCM1 stabilizes endothelial cell junctions via the small GTPase RAP1 and that CCM1/CCM2 interactions are required for this junctional stabilization [97]. The reasons for the prevalence of these vascular malformations in CNS endothelium are not yet understood, but may relate to the unique properties of the BBB and neurovascular unit.

### Cancer

Although cancerous brain cells emerge independently of the brain vasculature, the close functional relationships between CNS vessels and neural cells can lead to brain tumors that are highly vascularized and difficult to treat, primarily in the case of glioblastoma (reviewed in [98, 99]). Recently, several provocative studies provided evidence that tumor cells can mimic endothelial cell behavior to form vascular channels [100], and that tumor cells can even differentiate into endothelial cells that line blood vessels in glioblastoma [101–103]. It was further hypothesized that a tumor-initiating stem/progenitor cell might be the source of tumor-derived vascular cells ([101–103]; reviewed in [98]).

## Conclusions and unanswered questions

The cellular interactions and molecular signals critical for CNS vascularization and the formation of the neurovascular unit are being elucidated at an accelerating speed, and we are beginning to appreciate the significance of defective vascular development for the emergence of CNS pathologies. Yet, it is likely that additional signaling mechanisms and interactions remain to be identified before we will fully understand how the cross-talk of neural and vascular cells regulates blood vessel ingression into the CNS and the formation of a fully functional BBB. Ultimately, understanding these signaling pathways will reveal how a defective neurovascular unit impacts on CNS function during aging and in neurological disease. It will also benefit the development of new therapeutic strategies aimed at restoring or improving vascular supply to ischemic retina and brain in diseases such as diabetic retinopathy, age-related neurodegeneration and stroke. In the mature brain, neuronal activity stimulates changes in blood flow that can be measured by fMRI, but how this flow information is sensed and leads to structural changes in blood vessels is largely unknown and therefore requires further research. Finally, the specific features of the tumor microenvironment that promote the differentiation of tumor cells into endothelium, and the functional consequence of this transdifferentiation for glioblastoma remain to be determined.
